# ss-TEA: Entropy based identification of receptor specific ligand binding residues from a multiple sequence alignment of class A GPCRs

**DOI:** 10.1186/1471-2105-12-332

**Published:** 2011-08-10

**Authors:** Marijn PA Sanders, Wilco WM Fleuren, Stefan Verhoeven, Sven van den Beld, Wynand Alkema, Jacob de Vlieg, Jan PG Klomp

**Affiliations:** 1 Computational Drug Discovery Group, Radboud University Nijmegen Medical Centre, Geert Grooteplein, Nijmegen, The Netherlands; 2 Department of Molecular Design and Informatics, MSD, Molenweg, Oss, The Netherlands

## Abstract

**Background:**

G-protein coupled receptors (GPCRs) are involved in many different physiological processes and their function can be modulated by small molecules which bind in the transmembrane (TM) domain. Because of their structural and sequence conservation, the TM domains are often used in bioinformatics approaches to first create a multiple sequence alignment (MSA) and subsequently identify ligand binding positions. So far methods have been developed to predict the common ligand binding residue positions for class A GPCRs.

**Results:**

Here we present 1) ss-TEA, a method to identify specific ligand binding residue positions for any receptor, predicated on high quality sequence information. 2) The largest MSA of class A non olfactory GPCRs in the public domain consisting of 13324 sequences covering most of the species homologues of the human set of GPCRs. A set of ligand binding residue positions extracted from literature of 10 different receptors shows that our method has the best ligand binding residue prediction for 9 of these 10 receptors compared to another state-of-the-art method.

**Conclusions:**

The combination of the large multi species alignment and the newly introduced residue selection method ss-TEA can be used to rapidly identify subfamily specific ligand binding residues. This approach can aid the design of site directed mutagenesis experiments, explain receptor function and improve modelling. The method is also available online via GPCRDB at http://www.gpcr.org/7tm/.

## Background

G-protein coupled receptors (GPCRs), also known as 7 transmembrane receptors, represent a large superfamily of proteins in the human genome and are responsible for the transduction of an endogenous signal into an intracellular message, which triggers a response in many different physiological pathways. The structural architecture and chemo-mechanical concept of G-protein coupled receptors can be seen as an evolutionarily success as witnessed by the large amount of family members and diversity of applications in biological processes [1].

Not surprisingly, an increasing number of these GPCRs is the subject of investigation as targets in drug discovery. Historical drug discovery approaches have identified GPCRs as a successful drug target, since 25-50% of the drugs currently on the market interact with a GPCR [1,2].

In humans, the family of 7 transmembrane receptors is represented by approximately 900 members which can be divided in several classes based upon standard similarity searches [3-5].

Recently there has been a reclassification of receptors according to the GRAFS system which has the following groups: glutamate, rhodopsin, adhesion, frizzled/taste2, and secretin [6]. From the structural and functional viewpoint the rhodopsin-like family, also known as the class A receptors, is the largest and best studied family [6].

Receptors from different families are very diverse [1,5,7], but can all be characterized by the presence of seven structurally conserved alpha helices, which span the cell membrane. Most GPCRs couple to a G-protein complex upon ligand binding, resulting in the dissociation of the alpha subunit from the beta and gamma subunit. The final signal depends on the alpha subunit of the G-protein (G_αi_, G_αs_, G_αq/11_, G_α12/13_) which is activated and is presumed to be receptor and ligand dependent [8-12]. The non olfactory Class A receptors recognize a large variety of ligands including photons [13], biogenic amines [14], nucleotides [15], peptides [16], proteins [17] and lipid-like substances [18-21]. Most ligands are believed to bind fully or partly within the transmembrane bundle and to trigger signaling through a conserved canonical switch [9]. The assumption that similar molecules bind to similar receptors [22] and that small molecules bind within the upper part of the transmembrane helices, similar to 11-cis retinal in bovine rhodopsin, carazolol in the human beta adrenergic receptor 2, timolol in the turkey beta adrenergic receptor 1 and ZM-241385 in the human adenosine A2 receptor, gives rise to the application of pattern recognition analysis on multiple sequence alignments of those helices or parts thereof to identify ligand binding residues. It has also been shown that for some receptors which bind large proteins, like the luteinizing hormone receptor (LHR), low molecular weight (LMW) compounds can be designed which bind in between the TM-bundle and modify signaling [23,24], suggesting that the same pattern detection techniques could be used for those receptors as well.

Structure based drug design strategies often rely on high resolution information derived from protein crystal structures. Elucidating GPCR structures at atomic resolution remains difficult and has only been successful for a small set of receptors so far (bovine rhodopsin [25], squid rhodopsin [26], human beta-2-adrenergic receptor [27], turkey beta-1-adrenergic receptor [28] and the human A2A adenosine receptor [29]). These structures have been extremely helpful for understanding the function and ligand binding properties of class A receptors and are a major step forward towards rational drug design in this class of receptors. However, understanding the differences in for example agonist and antagonist binding or extrapolating structural information on a small subset of GPCRs to evolutionary distant receptors remains problematic and perhaps may only be solved as more structures become available [30]. As long as this information is limited there will be a need for comparative methods to explain the structural and functional differences between GPCRs.

With the recent genome sequencing efforts, more and more data becomes available to perform comparative modelling. Currently, data on 51 species is available in ensemble [31] (release 56) enabling the large scale comparison of sequences within and across species. Methods to mine sequence data and identify structurally and functionally important residues have been developed. For example, in 1996 Lichtarge introduced the evolutionary trace method to calculate the conservation of a residue in each trace of a phylogenetic tree [32]. In 2004 Oliveira et al. introduced the entropy variability plot and showed that the location of the aligned residue positions in these plots correlate to structural characteristics [33]. Based on a similar concept as the entropy variability plot Ye et al. introduced the two entropy analysis (TEA) in 2006 to identify structural and functional positions in the transmembrane region of class A GPCRs [34].

Here we present subfamily specific two entropy analysis (ss-TEA), the first method to identify the ligand binding residues on subfamily level. In contrast to the previously published methods ss-TEA is able to discriminate between subfamilies and able to identify the approximately five residues that are involved in ligand binding for each individual subfamily of the class A GPCRs. ss-TEA is predicated on high quality sequence information deduced from a multiple sequence alignment (MSA) which was generated by extracting species homologues of the class A non olfactory GPCR sequences with a method reported here. This new MSA is characterized by a more complete set of species orthologs which improves the subfamily definition and results of ss-TEA. Receptor specific sets of ligand binding residues, generated by ss-TEA, improve the understanding of receptor ligand interactions and the design of mutagenesis experiments, and guide the process of homology modelling.

## Results & Discussion

### Sequence retrieval & Alignment

Using a template set of 286 human GPCR sequences, a BLAST search was performed to retrieve non olfactory class A GPCR sequences. This resulted in 20111 sequences originating from 1941 species. An alignment of the transmembrane helices was obtained by gap free alignment of all retrieved sequences using HMM models of the TM domains. Subsequent removal of sequences with low HMM scores resulted in a MSA of 13324 class A GPCR sequences. 33 of the 1941 species contained over 100 class A non olfactory GPCR sequences and were deposited in a database and used for further analysis. The resulting multiple sequence alignment (MSA) comprises 6876 sequences of which 4816 sequences originate from Ensembl and 2060 from Swissprot and TrEMBL. For all aligned helices in the database, it can be shown that the overlap with the predicted helices in Swissprot is over 90% for 90% of the TM sequences and that almost no helices can be found which have less than 75% overlap (Additional file 1, Appendix 2). Due to the gap free alignment procedure of TM domains only those regions are subject to further analysis, loop regions will be omitted and anomalies in helix architecture, i.e. proline induced kinks will not be addressed.

**Figure 1:**
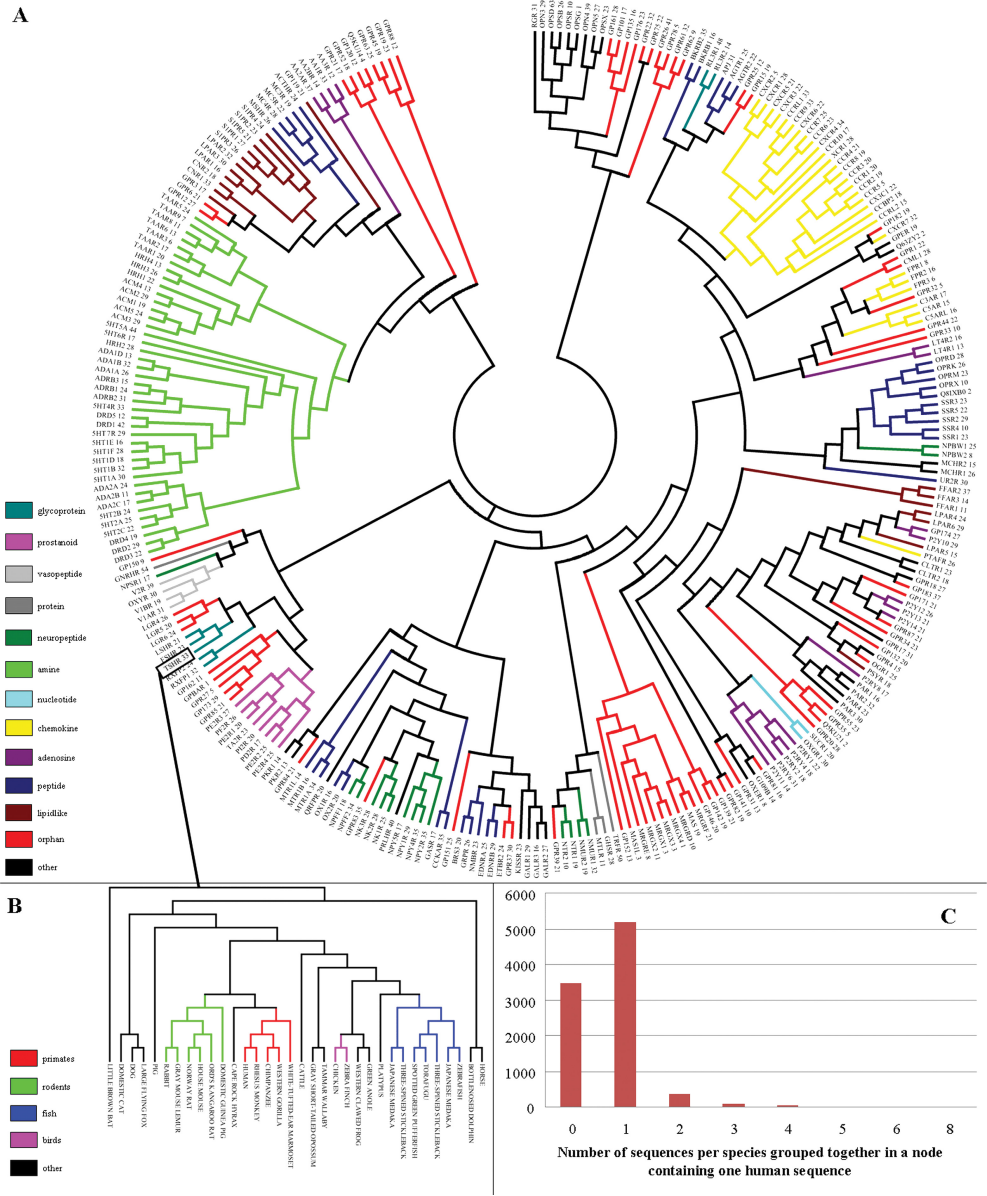
**Phylogenetic tree of all GPCR sequences**. A: Visualization of the human entries from the hierarchical tree constructed from the MSA of the TM domains from all sequences in the database. The number indicated after the receptor name equals the number of sequences which are grouped together in the visualized node. Leafs are colored according to the IUPHAR [36] family definition. B: Detailed view of the hierarchical tree of the branch including the human thyroid stimulating hormone receptor 1 with the leafs colored according to phylogenetic relatedness. C: Distribution of the number of sequences per species grouped together in a node containing one human receptor sequence. The number of missing receptor sequences was calculated with the assumption that each human receptor has one ortholog in each species.

From the distance matrix of all 6876 sequences a hierarchical tree was constructed. A visualization of all human entries of this tree is depicted in Figure 1A. The number of sequences from all other species, which can be grouped together with a human entry by collapsing a node, is indicated behind the receptor name. Phylogenetic analysis between human and mouse indicated that most human GPCRs have one ortholog in mouse [35]. Since most of the 33 species in our alignment are mammals with an evolutionary distance to human comparable to mouse (Additional file 1, Appendix 3), it is to be expected that one ortholog from every species can be grouped together with every human receptor. Exceptions to expected 1-1 ortholog pairs will be receptors that have been subject to gene expansion or have become pseudogenes. Examples include the MAS-related G-protein coupled receptors in which gene expansion has occurred, and the GNRHR and 5HT5A receptors which have pseudogenes in human [36]. Figure 1B shows the distributions of species sequences grouped together in a node with the thyroid stimulating hormone receptor (TSHR). Figure 1C displays that in most cases one sequence per species is grouped together in a node containing only one human sequence, suggesting that these are orthologs of this human receptor.

### Subfamily definition

**Figure 2:**
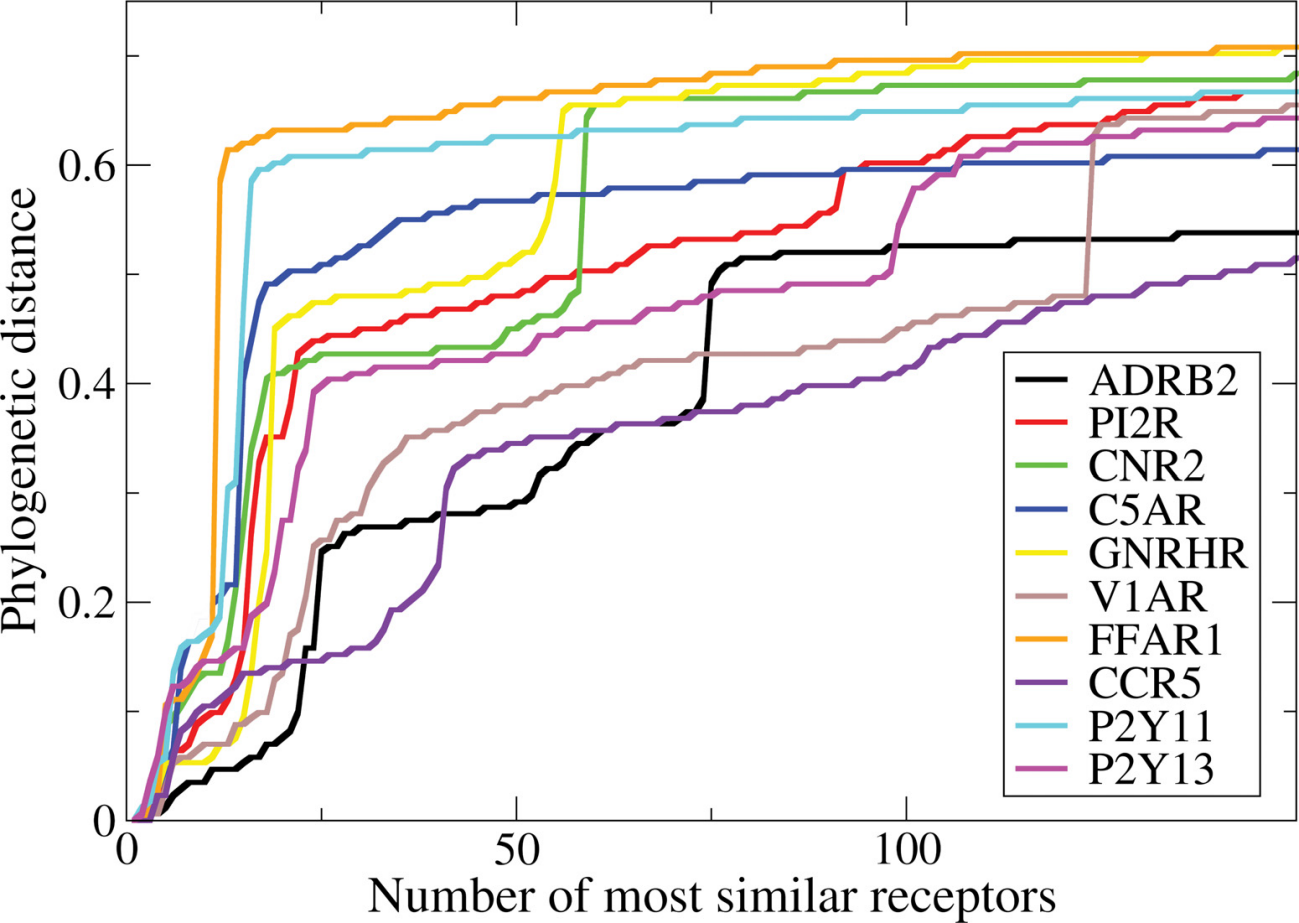
**Phylogenetic distance towards the human receptor as a function of the xth most similar receptor for the 10 receptors in the test set**.

To identify ligand binding residues we use a score composed of two entropy values. The underlying hypothesis for this score is that the ligand binding residues are conserved within a subfamily but not across all GPCRs. The residues which are conserved amongst all GPCRs are likely to be structurally important and can be easily identified by a low entropy value for all GPCRs. The size and variability of the subfamily should ensure that apart from structurally important residues only ligand binding residues are conserved within the subfamily. Phylogenetic distance is a measure for the sequence conservation in a subfamily. Figure 2 shows that most of the human receptors in our test set have small phylogenetic distances in subfamilies with sizes towards ~20 sequences. A subfamily of ~20-60 receptors contains homologous receptors (Figure 1) with slightly larger phylogenetic distances.

It is impossible to conclude whether or not ligand binding residues are conserved in a subfamily based on solely phylogenetic distances. Important aspects to consider in subfamily selection are that the receptors in a subfamily must bind to relatively similar ligands ensuring evolutionary pressure on the conservation of the residue positions involved in ligand binding, and that evolutionary distances are large enough to observe different amino acid usage amongst residue positions which are not involved in maintaining the structural architecture of the GPCR, signal transduction or ligand binding. We have therefore chosen to calculate the entropy values of all subfamilies with at least 50 and at most 300 sequences.

### Reference set

**Figure 3:**
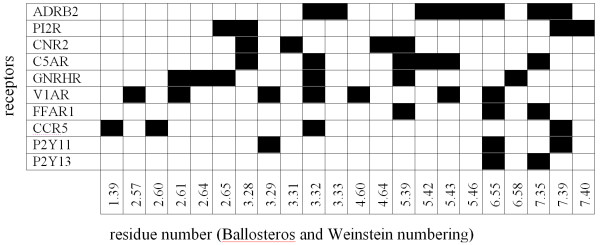
**Heatmap of reference residues sorted on position and receptor**. Ligand binding residues are colored black.

Site directed mutagenesis experiments offer a tool to investigate the function of specific residues in receptors. These experiments have helped to identify residues related to the signal transduction pathway as well as residues involved in ligand binding in GPCRs. Extracting this information from such experiments can however be very complicated, especially if ligands are compared which use different signaling pathways or when agonist are compared to antagonists. Antagonists only have to block active sites and this can be done via interactions with arbitrary residues. Agonists have to trigger certain responses and it is possible that ligands bind to different residues to trigger different responses. Another important aspect in the interpretation of mutation data is to separate direct from indirect effects. Mutations on the membrane facing side of a helix will for example very likely not affect ligand binding in a direct manner, but are more likely to have an influence due to distortion of the secondary structure. We have used site directed mutagenesis data described in literature, to the best of our knowledge, to compile a reference set of ligand binding residues for 10 selected receptors. This reference set consists of 47 residues located at 22 different positions (Figure 3).

### Ligand binding residue prediction

**Figure 4:**
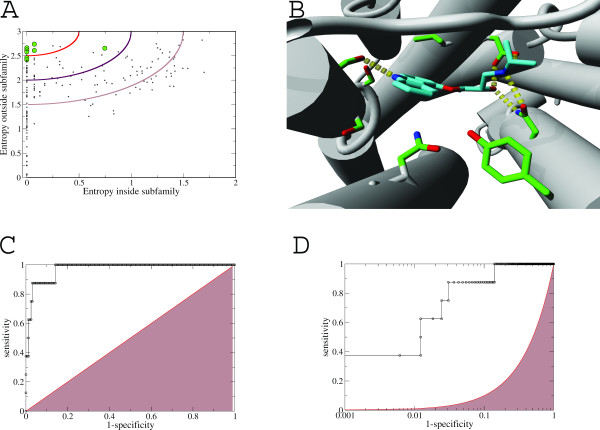
**Residue selection for the ADRB2 receptor**. A: Plot of the entropy within the ADRB2 receptor subfamily versus outside the subfamily. Lines are drawn at equal score and residues disrupting ligand binding upon mutation are colored green. B: Crystal structure of ADRB2 co-crystalized with carazolol (pdbid: 2RH1), residues disrupting ligand binding upon mutation are colored green. C: Receiver Operator Characteristic (ROC) curve showing the ability of ss-TEA to select ligand binding residues compared to random selection. D: ROC curve with logarithmic x-axis.

Prediction of ligand binding residues as performed by for example evolutionary trace, TEA and Multi-RELIEF is limited to the family level and results in a common description of the structurally important residues and ligand binding pocket. Analyses of the charged aspartate 3.32 in the amine receptors and lysine 7.33 in the opsins, known to be crucial for ligand binding from crystallography, show remarkable conservation characteristics. In both cases the residue is fully conserved inside the family and only rarely observed outside. This suggests that ligand binding residues can be identified by comparing the conservation level of a residue position within a subfamily to the conservation at this same position for all sequences outside this subfamily. In Figure 4A the two entropy values reflecting both observations are plotted for the ADRB2 receptor subfamily, which also includes the human receptors ADRB1 and ADRB3. The residues shown to disrupt ligand binding are colored green and are found in the upper left corner as expected. The distance of each residue to this upper left corner is used to rank the residues and used to evaluate the performance of ss-TEA. In Figure 4B the crystal structure of the ADRB2 receptor, co-crystalized with carazolol (pdbid: 2RH1) is visualized with the residues disrupting ligand binding colored green. The receiver operating characteristic (ROC) curves in Figures 4C and 4D, plotted with linear and logarithmic x-axis, show the improved ranking of residues according to ligand binding likelihood compared to random ranking. The area under the semi-logarithmic curve (Figure 4D) was used for further analysis because it puts more emphasis on correctly predicted ligand binding residues in the early phase of the recovery curve. It is typically in this region where performance needs to be outstanding, since many modeling approaches rely heavily on the correct assignment of only a limited number of ligand binding residues.

Table 1 shows that the mean area under the semi-logarithmic curve of the theoretically optimal ranking and ss-TEA are both 1.9. ss-TEA has the highest score in 7 out of 10 cases compared to the theoretically optimal ranking with the 4 highest scores out of 10 cases. A more realistic example is given by the comparison with the multi-RELIEF + 3d contacts method, which was reported to be the best performing method amongst several state-of-the-art methods [37]. The average pROC AUC of multi-RELIEF is 1.32 if the 22 reference residues are top ranked (see Methods section). ss-TEA gains 0.4 in the pROC AUC compared to multi-Relief in this situation and 0.5 if all residues are taken into account. It is also noteworthy that ss-TEA outperforms multi-Relief for all individual reference receptors except V1AR.

**Table 1:** Area under the semi logarithmic receiver operator curve (pROC AUC) of different rankings of residues for different targets

Target	Reference set (Ballosteros & Weinstein numbering scheme)	Multi-Relief	ss-TEA	Theoretically optimal (top-ranked)	Multi-Relief (top-ranked)	ss-TEA (top-ranked)
ADRB2	3.32, 3.33, 5.42, 5.43, 5.46, 6.55, 7.35, 7.39	1.2	**1.4**	**1.9**	1.6	1.6
PI2R	2.65, 3.28, 7.39, 7.40	1.0	**1.7**	1.5	1.4	**1.8**
CNR2	3.28, 3.31, 4.64, 5.39	0.7	**1.5**	1.6	1.1	**1.8**
C5AR	3.28, 3.32, 5.39, 5.42, 5.43, 7.35	1.1	**1.5**	**2.1**	1.5	1.7
GNRHR	2.61, 2.64, 2.65, 3.32, 5.39, 6.58	1.4	**1.8**	1.7	1.7	**1.9**
V1AR	2.57, 2.61, 3.29, 3.32, 4.60, 5.43, 6.55	**1.7**	1.3	1.8	**1.9**	1.5
FFAR1	5.39, 6.55, 7.35	0.9	**2.1**	**2.2**	1.3	**2.2**
CCR5	1.39, 2.60, 3.32, 7.39	1.1	**1.5**	1.7	1.6	**1.9**
P2Y11	3.29, 7.39, 6.55	1.5	**1.8**	1.9	1.8	**2.0**
P2Y13	6.55, 7.35	1.0	**2.1**	**2.2**	1.4	**2.2**

Average	22 res total	1.2	**1.7**	**1.9**	1.5	**1.9**

Top scoring methods are indicated in bold.

Three distinct receptors (ADRB2, CCR5 and GNRHR) which use different residue positions to bind ligands (see Figure 3) have been selected as an example to illustrate the advantage of the subfamily specific approach of ss-TEA.

**Figure 5:**
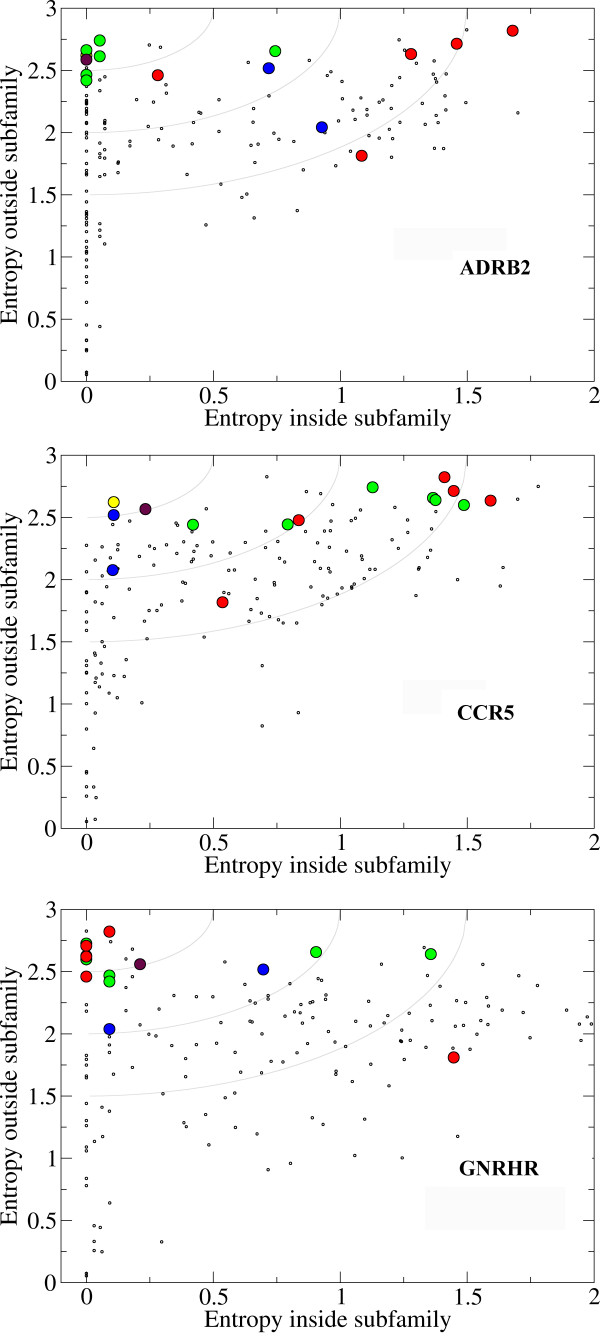
**ss-TEA plots of ADRB2, CCR5 and GNRHR respectively**. Ligand binding residues of the ADRB2 receptor are colored green, CCR5 receptor: blue, GNRHR receptor: red, ADRB2 and CCR5 receptor: yellow and of all three receptors: maroon.

Figure 5 shows in green the residues involved in ligand binding to the ADRB2 receptor only. Likewise, the ligand binding residues for CCR5 and GNRHR are colored blue and red respectively. Residue 7.39 is important for ligand binding in both the ADRB2 and CCR5 receptors and is colored yellow, while position 3.32, colored maroon is a ligand binding residue for all three receptors. Figure 5 shows that green residues are mainly located in the upper left corner of the ADRB2 plot, while the red and blue residues are positioned more to the right. Similar distributions are observed for the blue and red residues in the CCR5 and GNRHR plot respectively, clearly illustrating that the selection of residues by ss-TEA are subfamily specific.

**Figure 6:**
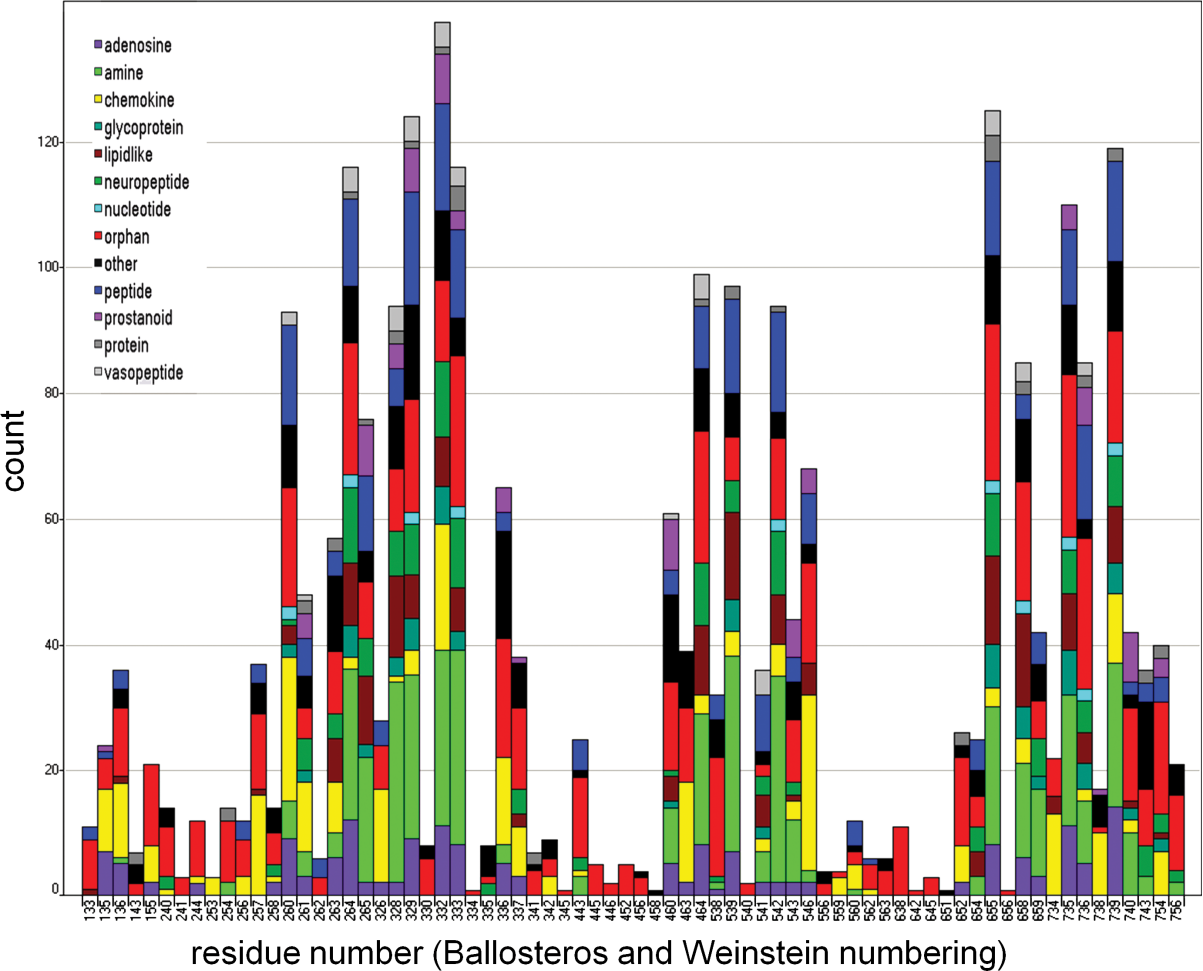
**Distribution of residue positions scoring amongst the top 10 based on ss-TEA for all human receptors**. Bars are colored according to the iuphar family description.

Analyses of the highest ranked residues for all individual human receptors identify subfamily ligand binding characteristics. Determination of the top 10 scoring residues for all human receptors visualized in Figure 6 and colored according to the IUPHAR family definition [36], shows that there is no generic ligand binding mode for class A GPCRs since none of the positions is scored amongst the top 10 for more than 50% of the in total ~300 human receptors. Furthermore it can be seen that that helix I is rarely important for ligand binding, as also observed in the available crystal structures. Even so, some orphan, adenosine and chemokine receptors are characterized by conservation patterns for residues in this helix and might bind ligands with residues from this helix. In addition, the amine receptors can be characterized by the importance of helix three in ligand binding.

However the comparison of individual receptors within a receptor family also reveals interesting differences in ligand binding behavior. This is illustrated by e.g. position 3.32, which is well conserved in about 50% of all subfamilies, including the aminergic receptors and a subset of the adenosine receptors. For the aminergic receptors it has been proposed that this aspartate is crucial for ligand binding due to its interaction with the positively charged nitrogen of the basic amines, a hypothesis which is confirmed by the crystal structures of ADRB2 and ADRB1. For other receptors this same position is thought to be important for ligand binding involving different amino acids. For example, AA2AR receptor has a conserved valine at position 3.32. Mutation of this valine to alanine or aspartate disrupts ligand binding and illustrates the importance of this conserved valine for this receptor [38]. Position 3.32 ranks at position 45 in the AA1R subfamily, while it ranks at position 11 the AA2AR subfamily suggesting a less important function for the valine in the AA1R receptor, which is indeed confirmed by site directed mutagenesis [38].

Interestingly, receptors with endogenous ligands which completely or largely bind to the N-terminus and/or extracellular loops also demonstrate subfamily specific conservation of residues at the extracellular side of the transmembrane helices. It is remarkable, for example, that 8 of the top 10 ranked residues for the luteinizing hormone receptor are in fact pocket residues. Also noteworthy is that Asp2.64, known to interact with the endogenous ligand [39], is ranked 3^rd^.

## Conclusions

We have introduced an alignment methodology to create a large multiple sequence alignment of the transmembrane domains of class A non olfactory GPCRs from multiple species. We also introduced a new method to identify ligand binding residues from a MSA, named ss-TEA, and demonstrated the advantage of this new method in combination with the new MSA for the selection of ligand binding residues. The results show the advantage of receptor specific residue selection compared to receptor class specific selection, as well as an improved residue selection for 9 of the 10 reference sets in comparison to the state-of-the-art method Multi-Relief. The large MSA including sequences of multiple species allows us to compare receptors with high sequence similarities and more identical ligand binding profiles which results in a better understanding of the characteristics of those receptors. If more sequence data becomes available for more species, larger alignments can be made, which could possibly even explain differences between close homologs. Our alignment in combination with the residue selection method described here can be used to quickly identify ligand binding residues. This can subsequently be used to design site directed mutagenesis experiments, explain receptor function and improve modelling. The ss-TEA predictions for class A GPCRs can be accessed via GPCRDB at http://www.gpcr.org/7tm/.

## Methods

**Figure 7:**
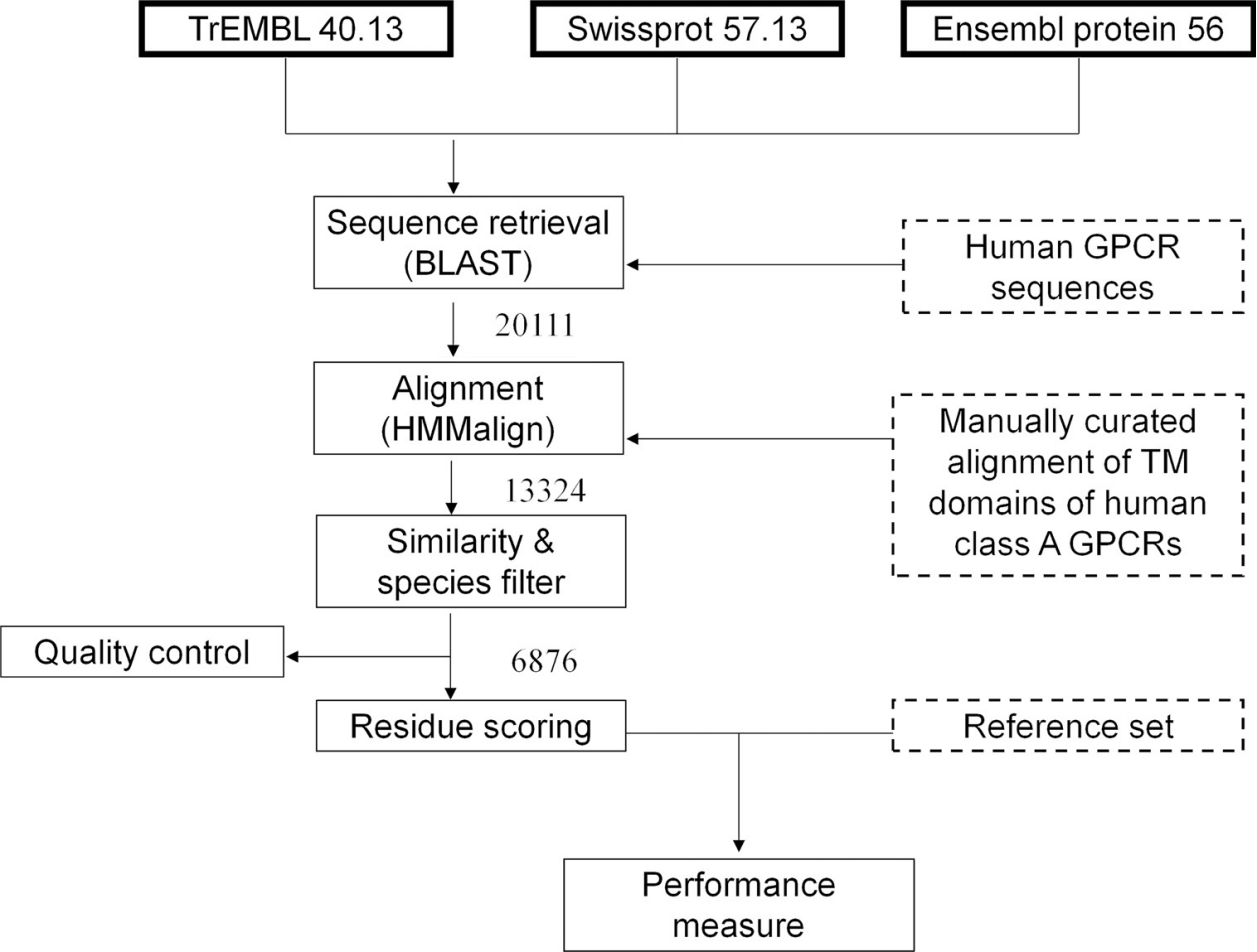
**Schematic flowchart of the methodology to create the alignment, score the residues and evaluate the performance of the residue selection method**. Publicly available data sources are indicated with a bold border style, in-house data with dashed a border style and methods with a normal border style. The numbers indicate the number of sequences which is used at each step.

Our approach makes use of different input sources which are connected via algorithms as outlined in Figure 7. All steps will be outlined and discussed in sequential order below.

### Sequence retrieval

The first step in our approach is to extract GPCR sequences for different species from available data sources. To obtain sequences we performed a BLAST [40] search with 286 manually curated query sequences from human class A non-olfactory GPCRs against Swissprot, Ensembl and TrEMBL. All query sequences were blasted against Swissprot 57.13 [41,42], Translated EMBL (TrEMBL) 40.13 [42,43] and Ensembl Protein 56 [31], using the BLOSUM62 scoring matrix, an expected cutoff of 10 and word size 3. Furthermore, a gap opening penalty of 11 and a gap extension penalty of 1 were used. Finally, we selected all sequences with an e-value < 0.01, subject length identity > 25%, alignment identity > 40% and a minimal query length of 20 amino acids.

### Numbering Scheme and MSA boundaries

The aim of the Multiple sequence alignment (MSA) is to reflect a structural alignment and therefore the loop regions and termini of all receptors were omitted, since these are not structurally conserved. The positions included in our MSA according to the Ballosteros and Weinstein numbering scheme [43] are: 1.33-1.56 for TM1; 2.40-2.65 for TM2; 3.25-3.51 for TM3; 4.43-4.64 for TM4; 5.38-5.63 for TM5; 6.37-6.59 for TM6; and 7.34-7.56 for TM7. The pocket is defined by 28 residues which are directed towards the intramembrane cavity in the upper part of the transmembrane domains in the available crystal structures. The residues defined as pocket per transmembrane region are: 1.35, 1.39, 1.42, 1.46 for TM1; 2.57, 2.58, 2.61, 2.65 for TM2; 3.28, 3.29, 3.32, 3.33, 3.36 for TM3; 4.56 for TM4; 5.38, 5.39, 5.42, 5.43, 5.46 for TM5; 6.44, 6.48, 6.51, 6.52, 6.55 for TM6 and 7.35, 7.39, 7.43, 7.45 for TM7.

### Alignment

**Figure 8:**
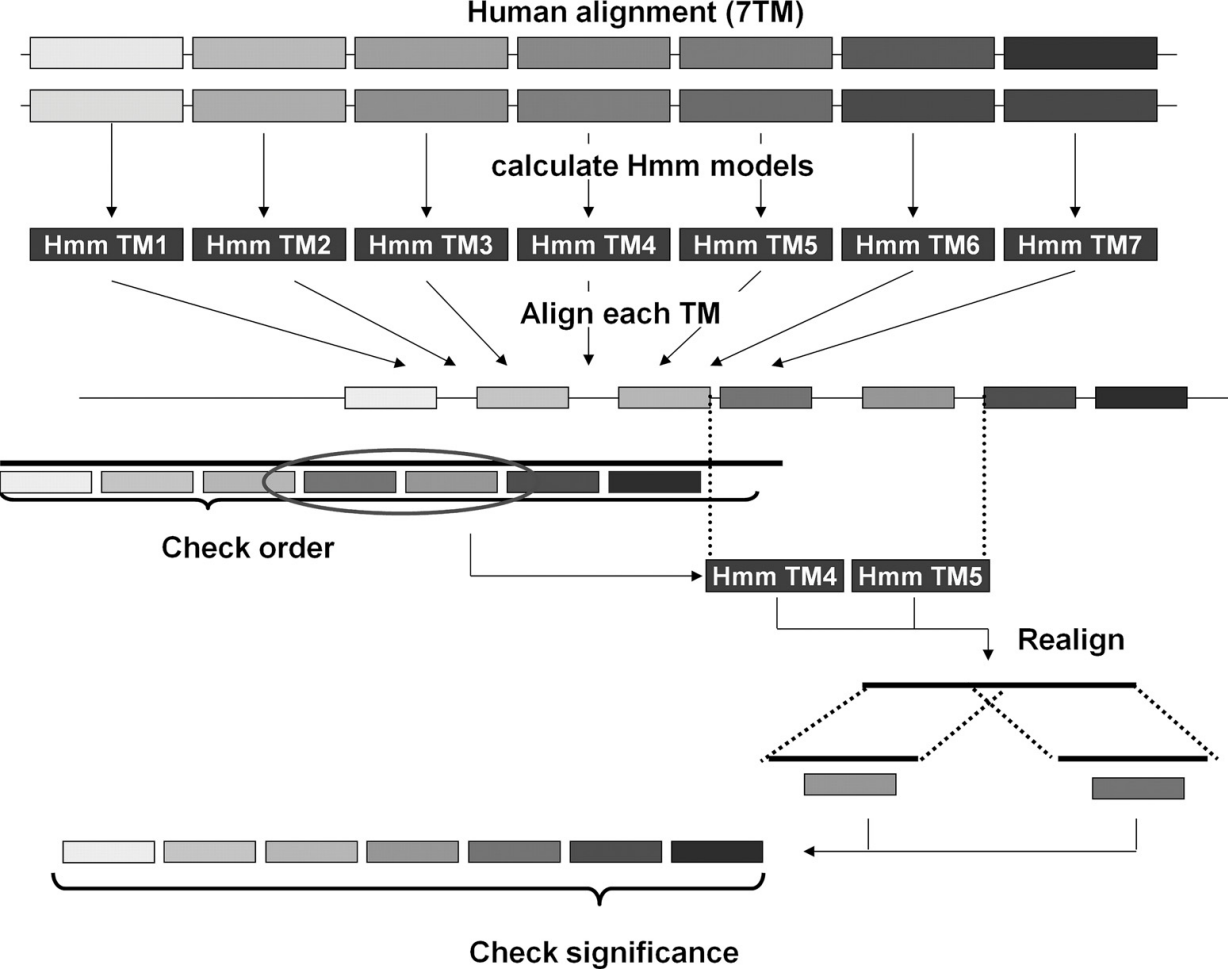
**Schematic representation of the alignment procedure**. First a HMM model is calculated for each TM domain from a manually curated alignment of the 7TM domains of 286 human GPCRs. Each HMM model is subsequently aligned to each GPCR sequence after which the ordering of the aligned helices is checked. In case of an incorrect ordering realignment is performed on smaller parts of the sequence. Finally the significance of each aligned helix is checked.

The available GPCR crystal structures have shown that all helices can be structurally aligned without introducing gaps in the sequence alignment. For this reason a manually curated gap-free alignment of the TM domains of the human class A non-olfactory GPCR sequences was created and used to construct a Hidden Markov Model (HMM) for each separate helix, using HMMbuild (HMMER [44] 2.3.2 (Oct 2003)) with default settings. Subsequently each hmm model of each helix was aligned against all extracted sequences from the previous step, without allowing the introduction of gaps, using HMMalign (HMMER [44] 2.3.2 (Oct 2003)). Alignments which had an incorrect helix ordering were subsequently extracted and subject to realignment on a smaller part of the sequence. A typical example is the realignment of one helix on the sequence in between two correctly aligned neighbor helices (see Figure 8). As a final filter, all sequences with a low similarity score to the hmm model for over 4 out of 7 helices were discarded. A threshold of 4 was chosen, since a few annotated human sequences, e.g., the prostanoids, were shown to have weak patterns for up to 4 helices. The threshold for the similarity score of each individual helix was set after the compilation of artificial sequences with an identical amino acid distribution for each helix as in the manually curated alignment of human GPCRs. These artificial sequences were subsequently aligned to the previously built hmm model of the helix, and the threshold for the helix was set to the score at which 95% of the artificial sequences fails to pass. Low sequence quality may cause duplicate entries of the same receptor and species. To avoid these duplicates, all but 1 sequence, of all sets of sequences of an individual species which had less than 10 amino acids difference, were removed.

### Database

Incomplete sequencing of the genomes of many species causes bias towards certain receptor subfamilies. To prohibit such bias, all sequences of species with less than 100 amino acid sequences of GPCRs were removed from the MSA. All GPCR sequences of species of which at least 100 different sequences were obtained, were stored in a database and used in all analysis discussed below. To enable querying on a higher level than the individual sequences, a hierarchical tree of the phylogenetic distance matrix calculated from the alignment of all 7 TMs of all receptors was created, using the neighbor joining algorithm as implemented in clustalW [45] 2.0.11 with a 100 fold bootstrap. The sequences which group together at a node in this tree, a so called subfamily, can be queried for their properties.

### Residue selection

To perform knowledge based residue selections which reflect the likelihood of residues being involved in ligand binding, we added two Shannon entropy scores for each alignment position of each receptor to the database. One entropy value reflects the conservation of a position inside the subfamily (*E^in^*) while the other entropy reflects the conservation of this same position in all sequences which do not belong to this subfamily (*E^out^*). The Shannon entropy itself is given by:(1)

With(2)

*Number_ia _*is the number of sequences with residue type *a *at alignment position *i*. Others have already suggested that ligand binding residues can be obtained from both calculated entropy values [33,34]. Therefore we introduce one score which combines both calculated entropies.(3)

A final score for each residue position was calculated after evaluation of the score at multiple branches of the hierarchical tree using:(4)

where *j *reflects the number of sequences selected in the branch. To validate the performance we finally ranked all residues according to the score with the minimum scoring residue at rank 1.

### Reference Set

Site directed mutagenesis data is available for many GPCRs with different levels of detail depending on the research question. In this paper ten well studied and evolutionary diverse Class A GPCRs are used for which extensive site directed mutagenesis data exists as well as a binding model based on these data. For each of the receptors a reference set of residues crucial for ligand binding was compiled using the mutation data described in GPCRdb [5] and literature models of the binding mode. The choice of receptors from different branches of the sequence tree was made to emphasize the advantage of a method able to identify different ligand binding residues for different receptors and to show that the method does not have a bias towards certain subfamilies. The receptors in the reference set are; beta-2 adrenergic receptor (ADRB2) [27,46]; Prostacyclin receptor (PI2R) [47]; C5a anaphylatoxin chemotactic receptor (C5AR) [48]; Cannabinoid receptor 2 (CNR2) [49,50]; Gonadotropin-releasing hormone receptor (GNRHR) [51]; Vasopressin V1a receptor (V1AR) [24]; Free fatty acid receptor1 (FFAR1) [52]; C-C Chemokine receptor type 5 (CCR5) [53]; P2Y purinoceptor 11 [54] and 13 [55] (P2Y11, P2Y13). Residues that were not part of the pocket [56] were neglected as well as mutations which are debatable because of different effects using different ligands or because results were not consistent in different measurements. The final selection only includes residues with substantial effect on ligand binding. The A2A adenosine receptor was deliberately not used as a reference set in this study, since site directed mutagenesis data and the crystal structure suggest that there is no general, family conserved receptor binding pocket for the A2A adenosine receptor [29,38].

### Performance measure (Area Under the Log Curve)

The performance of our residue ranking method is assessed using the Area under the semi-logarithmic receiver operating characteristic (ROC) curve [57]. This method favors true ligand binding residues early in the recovery curve and is calculated using:(5)

Where *n *is the number of true ligand binding residues and *β_i _*is the false positive frequency corresponding to the point at which the *i*th true residue is found. *β_i _*is typically calculated as the fraction of false positives which is ranked higher than the *i*th true positive. The score of the pROC AUC corresponding to a random selection is 0.434 and is unbounded on the high side. A perfect ordering of ligand binding residues amongst 100 non ligand binding residues will for example score 2.0.

### Benchmark

To illustrate the advantage of subfamily specific ranking over generic ranking we compiled a theoretically optimal generic ranking of ligand binding residues. This ranking is created by ordering the residues of ten different receptors according to the number of receptors which use these positions for ligand binding. The ranking of positions used by the same number of receptors is arbitrary, potentially altering the results, although it is expected to have only a minor effect. Because the theoretically compiled optimal ranking includes information about the location of the pocket we also included this information in the ss-TEA and Multi-Relief method and scored the 22 residues included in the theoretically compiled optimal ranking prior to all other residues. The rankings which include this information will be indicated in this paper as top ranked. As a benchmark we compared our top ranking to both the theoretically compiled optimal ranking and Multi-Relief + 3d contacts top ranking [37] (Additional file 1, Appendix 1). Briefly, Multi-Relief takes a multiple sequence alignment and predefined subfamily ontology as input, then iteratively selects 2 subfamilies and optimizes a weight vector able to optimally separate the sequences from both [37]. The optimization of a single weight vector in the iterative process results in one vector able to discrimate between all provided classes. The weight of a residue in the Multi-Relief + 3d contacts method can be altered towards its local environment as obtained from recent crystal structures.

## Authors' contributions

WF and SV retrieved possible class A GPCR sequences from the described data sources. SvdB and SV created the multiple sequence alignment. MS developed the residue selection method and was responsible for the draft of the manuscript. JK, JdV and WA supervised the project and helped to draft the manuscript. All authors read and approved the final manuscript.

## Supplementary Material

Additional file 1 **Theoretically compiled optimal ranking and Multi-RELIEF ranking of the residues included**. Percentage of helix overlap between the generated alignment and predicted helix locations in swissprot. Phylogenetic tree of the species included in our sequence alignment. Click here for file
