# Supplementary material for: ss-TEA: Entropy based identification of receptor specific ligand binding residues from a multiple sequence alignment of class A GPCRs
Source: BMC Bioinformatics. 2011 Aug 10;12:332. doi: 10.1186/1471-2105-12-332 (PMC3162937; doi:10.1186/1471-2105-12-332)
Supplement: Additional file 1 — Theoretically compiled optimal ranking and Multi-RELIEF ranking of the residues included. Percentage of helix overlap between the generated alignment and predicted helix locations in swissprot. Phylogenetic tree of the species included in our sequence alignment. [file 1471-2105-12-332-S1.DOC]

Appendix 1

| Rank | Theoretically optimal ranking | Multi-Relief +3d contacts |
| --- | --- | --- |
| 1 | 3.32 | 3.32 |
| 2 | 6.55 | 3.29 |
| 3 | 5.39 | 2.61 |
| 4 | 7.35 | 2.57 |
| 5 | 7.39 | 2.64 |
| 6 | 3.28 | 3.33 |
| 7 | 5.43 | 7.39 |
| 8 | 2.61 | 6.55 |
| 9 | 2.65 | 3.28 |
| 10 | 3.29 | 7.35 |
| 11 | 5.42 | 2.60 |
| 12 | 1.39 | 4.60 |
| 13 | 2.57 | 7.40 |
| 14 | 2.60 | 6.58 |
| 15 | 2.64 | 5.42 |
| 16 | 3.31 | 5.43 |
| 17 | 3.33 | 2.65 |
| 18 | 4.60 | 5.39 |
| 19 | 4.64 | 5.46 |
| 20 | 5.46 | 4.64 |
| 21 | 6.58 | 3.31 |
| 22 | 7.40 | 1.39 |

Table 1: Ranking of residues according to a theoretically optimal ranking as well as according to the multi-RELIEF +3d contacts method. Residue numbers are colored according to the number of reference structures in which they are involved in ligand binding. Green: 5 receptors, Orange: 4 receptors, Red: 3 receptors, Blue: 2 receptors and Black: 1 receptor.

**Appendix 2**

Figure 1: Overlap of Transmembrane (TM) domains between the alignment of sequences in swissprot and our own multiple sequence alignment.

**Appendix 3**


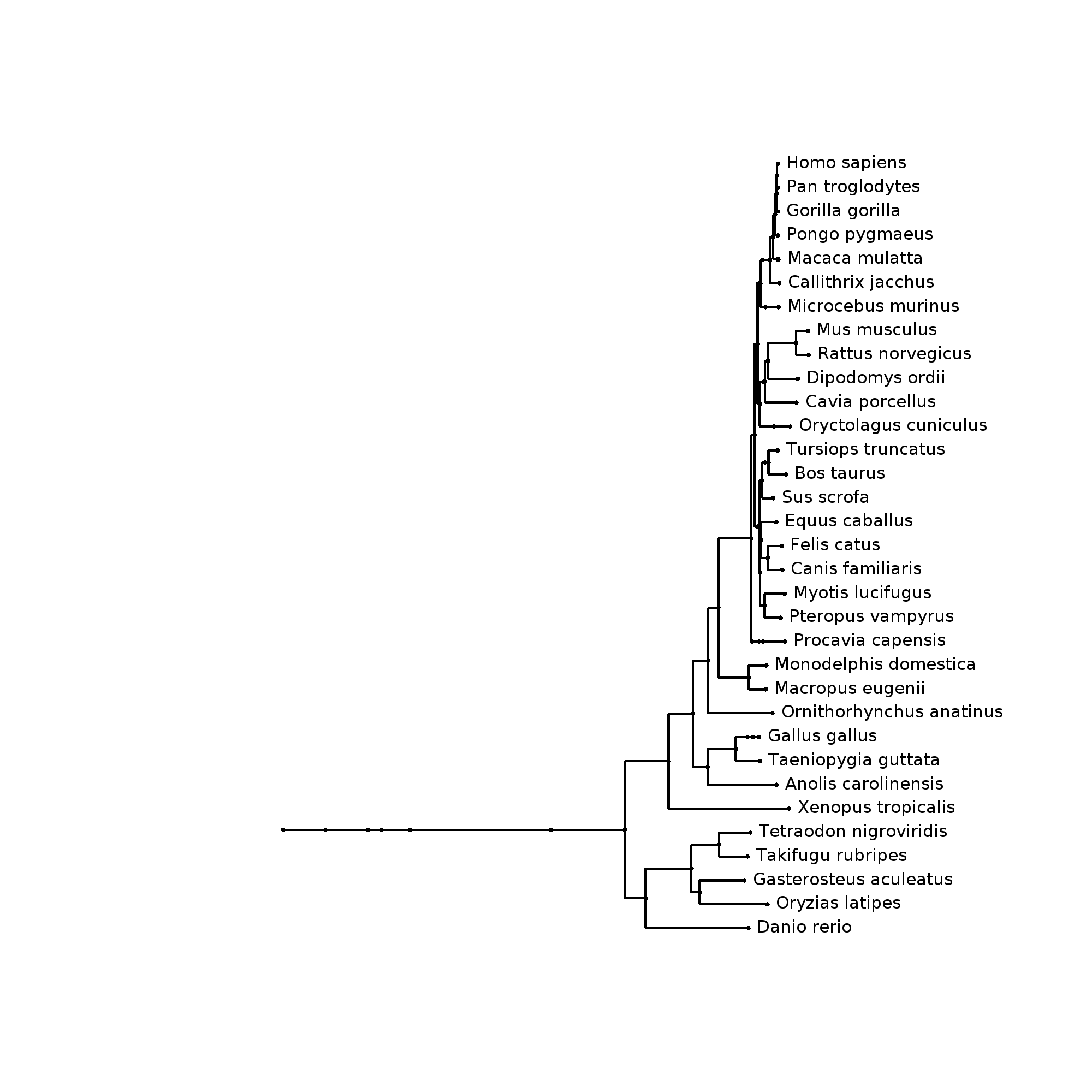


Figure 1: Phylogenetic tree of species included in our database as calculated by Ensembl.
